# Selection and Effect of Plant Growth-Promoting Bacteria on Pine Seedlings (*Pinus montezumae* and *Pinus patula*)

**DOI:** 10.3390/life14101320

**Published:** 2024-10-17

**Authors:** Francisco David Moreno-Valencia, Miguel Ángel Plascencia-Espinosa, Yolanda Elizabeth Morales-García, Jesús Muñoz-Rojas

**Affiliations:** 1 Consejo Nacional de Ciencias, Humanidades y Tecnología (CONAHCYT)—Group “Ecology and Survival of Microorganisms”, Laboratorio de Ecología Molecular Microbiana, Centro de Investigaciones en Ciencias Microbiológicas, Instituto de Ciencias, Benemérita Universidad Autónoma de Puebla, Puebla C.P. 72570, Mexico; franciscod.moreno@correo.buap.mx; 2 Centro de Investigación en Biotecnología Aplicada (CIBA), Instituto Politécnico Nacional, Ex-Hacienda San Juan Molino, Carretera Estatal Tecuexcomac-Tepetitla Km 1.5, Tlaxcala C.P. 90700, Mexico; 3 Grupo Inoculantes Microbianos, Facultad de Ciencias Biológicas, Benemérita Universidad Autónoma de Puebla, Puebla C.P. 72570, Mexico; yolanda.moralesg@correo.buap.mx; 4 Group “Ecology and Survival of Microorganisms”, Laboratorio de Ecología Molecular Microbiana, Centro de Investigaciones en Ciencias Microbiológicas, Instituto de Ciencias, Benemérita Universidad Autónoma de Puebla, Puebla C.P. 72570, Mexico

**Keywords:** forest species, phyto-stimulation, plant growth-promoting mechanisms, reforestation, speed germination

## Abstract

Forest cover is deteriorating rapidly due to anthropogenic causes, making its restoration urgent. Plant growth-promoting bacteria (PGPB) could offer a viable solution to ensure successful reforestation efforts. This study aimed to select bacterial strains with mechanisms that promote plant growth and enhance seedling development. The bacterial strains used in this study were isolated from the rhizosphere and endophyte regions of *Pinus montezumae* Lamb. and *Pinus patula* Schl. et Cham., two Mexican conifer species commonly used for reforestation purposes. Sixteen bacterial strains were selected for their ability to produce auxins, chitinase, and siderophores, perform nitrogen fixation, and solubilize inorganic phosphates; they also harbored genes encoding antimicrobial production and ACC deaminase. The adhesion to seeds, germination rate, and seedling response of *P. montezumae* and *P. patula* were performed following inoculation with 10 bacterial strains exhibiting high plant growth-promoting potential. Some strains demonstrated the capacity to enhance seedling growth. The selected strains were taxonomically characterized and belonged to the genus *Serratia*, *Buttiauxella*, and *Bacillus*. These strains exhibited at least two mechanisms of action, including the production of indole-3-acetic acid, biological nitrogen fixation, and phosphate solubilization, and could serve as potential alternatives for the reforestation of affected areas.

## 1. Introduction

The degradation of soils and the decline of native vegetation due to anthropogenic activities is an increasingly severe environmental issue [1]. Deforestation rates from 2015 to 2020 reached an estimated 10 million hectares per year, resulting in the loss of 420 million hectares of forest since 1990 due to land-use changes [2]. These trends, coupled with global concerns over climate change and biodiversity loss, emphasize the critical role of forest cover in mitigating air pollution, carbon sequestration, and preserving ecosystem services and habitats [3,4,5]. Reforestation holds significant potential for soil and water conservation, making the forestry sector a key driver for sustainable development [6]. The most impactful strategy to counteract climate change is the expansion, restoration, and management of forests [7], which also yields long-term benefits for biodiversity conservation [8]. Despite global reforestation programs aimed at mitigating these effects [2], the expected results have not been fully achieved [9]. The survival of forest seedlings is largely influenced by the introduction of non-native species, insufficient post-planting care, and the lack of stress tolerance studies on selected plant species [10]. In Mexico, forestry activities are primarily focused on the *Pinus* genus, which accounts for 60% of commercially valuable species [11]. This coniferous genus is distributed across 24 states, with two states in the North Central region, one in the West, one in the South, and two in the Central Gulf region standing out [12]. The economic importance of pine in Mexico is linked to its contribution to the country’s economy and its role in the Gross Domestic Product (GDP) of the forestry sector. Socially, its relevance stems from the communities living in forested areas who rely on the goods and services provided by pine forests [13]. Given the importance of pine species in Mexico, *Pinus montezumae* Lamb. and *Pinus patula* Schl. et Cham. are included in the technological packages currently applied for soil restoration and conservation in areas degraded by human activities. *Pinus patula* is widely used for timber and cellulose production due to its high productivity and adaptability to various abiotic conditions and non-forested soils [14,15]. *Pinus montezumae* has been successfully employed in several reforestation programs aimed at watershed protection and soil restoration [16].

An effective strategy to enhance plant survival during the adaptation phase is the application of microbial technology that supports plant growth and ensures successful transplantation [17]. Plant Growth-Promoting Bacteria (PGPB) inoculants have been developed due to their growth-promoting properties, offering both direct and indirect mechanisms of action, primarily in agricultural crops. PGPB are classified into two groups, symbiotic and free-living, based on their relationship with plants [18]. They are further categorized as extracellular or intracellular PGPB depending on their location within the plant [19,20]. Extracellular PGPB are found in the rhizosphere, rhizoplane, or spaces between root cortex cells, while intracellular PGPBs exist within the root cells [21,22,23]. Due to their plant growth-promoting effects, these beneficial microorganisms are often referred to as yield-increasing bacteria, plant health-promoting rhizobacteria, or nodule-promoting rhizobacteria, depending on their mode of action on plant metabolism [24]. PGPB also enhances soil water retention, helping to mitigate drought conditions to some extent [25]. PGPB are further classified based on their activities as follows: as biofertilizers, they increase the solubilization of minerals and fix nitrogen, making nutrients more accessible to plants; as phytostimulators, they produce phytohormones such as indole-3-acetic acid (IAA), abscisic acid, gibberellins, cytokinins, and ethylene [26,27]; and as biocontrol agents, they release a wide variety of antibiotics and antifungal compounds that protect plants from biotic stress, including siderophores, β-1,3-glucanase, chitinases, antibiotics, fluorescent pigments, and cyanide [28]. Furthermore, PGPB that produce 1-aminocyclopropane-1-carboxylate (ACC) deaminase, a critical enzyme, help reduce ethylene levels in plant roots, promoting increased root length and growth [29,30]. Lastly, as rhizoremediators, PGPB enhance plant growth by removing organic contaminants from the rhizosphere, and improving plant tolerance to salinity [31], metal toxicity [32], and drought through mechanisms such as exopolysaccharide (EPS) production [33,34], biofilm formation, and osmolyte reduction to prevent cellular moisture loss [35,36]. These mechanisms may act simultaneously and synergistically during different stages of plant growth. This biotechnological approach is environmentally friendly, with no adverse effects [37,38]. Furthermore, inoculating forest seedlings with PGPB in nurseries increases beneficial microbial populations in the plant’s rhizosphere. As the plant serves as a vehicle for reintroducing these microbes into the soil, it promotes early growth, reduces transplant stress, and enhances adaptation to the new environment [39].

PGPB have been isolated from various plant-associated environments, including the rhizosphere, endophytic, and epiphytic zones [40,41,42]. However, the isolation of bacteria with the potential to promote tree growth has been less extensively studied [43,44,45,46], despite its significant implications for the productivity of certain fruit crops and the reforestation of forested areas. Evidence suggests that there is significant host specificity in tree species when treated with these microorganisms, which may be influenced by local environmental and geographic conditions [47]. Thus, the mutualistic relationship between microorganisms and plant growth processes is critical for the successful establishment of nursery-grown seedlings in their new habitats. Root exudates are generally plant-specific and often serve as signals to facilitate affinity with particular microorganisms [10,48]. This occurs because plants can “select” their microbiome for beneficial bacterial colonizers, including those residing within plant tissues [49]. Soil degradation and the loss of native vegetation due to unsustainable human activities are escalating issues that impact geoecosystems in Mexico and worldwide. Effective solutions are needed, such as reforestation, which plays a vital role in soil and water conservation by sequestering carbon, improving soil fertility, regulating river flows, and creating favorable microclimates. This paper proposes, presents, and discusses a growth acceleration system for forest species through the isolation and selection of plant growth-promoting bacteria, aimed at enhancing the physiological and morphological traits of nursery seedlings. The expected outcome is an improvement in the establishment and survival rates of forest seedlings compared to the current cultivation methods. Additionally, the paper discusses the selection of bacterial strains based on their plant growth-inducing mechanisms and the growth-promoting effects observed when inoculated into *P. montezumae* and *P. patula* seedlings.

## 2. Materials and Methods

### 2.1. Bacterial Strains

Bacterial strains evaluated in this study were collected from the *P. patula* and *P. montezumae* rhizosphere and endophytic zones in the forested regions of Tlaxcala, Mexico. A total of 35 strains from *P. patula* and 67 strains from *P. montezumae* were isolated and cataloged. The naming convention for these strains included a sequential number based on their isolation order, the collection site, and the tree species from which they were collected [10]. This work is a continuation of our previously conducted study [10]; however, a brief description of how the isolation of the strains studied was carried out is provided below. The site for collecting plant material was chosen due to the natural distribution of *P. montezumae* and *P. patula*. Two collections of *P. montezumae* were formed in Malinche National Park, at four-week intervals between August and September 2016. Additionally, *P. patula* was sampled in the Sierra de Tlaxco–Caldera–Huamantla region in November of the same year. A field inspection was conducted to identify healthy seedlings with no visible damage or disease. Each collected tree was evaluated for dendrometric and soil moisture measurements, and the entire seedlings were transported to the laboratory for the isolation of rhizospheric and endophytic colonies under aseptic conditions.

Rhizospheric bacteria were isolated from the soil most adhered to the seedling roots. A suspension of rhizospheric soil was prepared by immersing the roots in sterile distilled water (1:10 *w*/*v*) and homogenizing the sample. Serial dilutions were performed at a 1:7 dilution factor, and 200 µL of the sample from each dilution were spread on two different culture media using the plate spreading technique. Endophytic bacteria were isolated from inside the root. The root surface was sterilized by immersing it in 96% ethanol for 5 min, followed by 6.25% NaOCl for 10 min, and then it was rinsed thoroughly with sterile distilled water. The plant material was immersed in a 0.1 M aqueous solution of MgSO_4_ and ground in a porcelain mortar [50]. The resulting macerate was then inoculated in triplicate [51,52] on Mannitol Yeast Extract Agar (pH 7) [53] and Nutrient Agar (pH 6.8) [54]. All plates were subsequently incubated at 28 °C for 5 days. Different colonies that grew on the plates were isolated, purified, and stored in a glycerol–water solution (1:4) at −20 °C for preservation and future use.

### 2.2. Characterization of the Isolated Bacteria Based on Their Mechanisms of Action

#### 2.2.1. Indole Acetic Acid Production and Biosynthetic Pathway

Bacterial cells were induced by synthetic L-tryptophan (Sigma-Aldrich Production GmbH, Buchs, Switzerland), suspended in a solution of 10% methanol/0.05% acetic acid and then sonicated and centrifuged at 12,000 rpm for 20 min. The supernatant was filtered through a 0.22 µm PVDF membrane before analysis by reverse phase high performance liquid chromatography and mass spectrometry (RP-HPLC–MS/MS). The analysis was performed using a Shimadzu Nexera HPLC system coupled with a TRAP 3200Q mass spectrometer (SCIEX, Framingham, MA, USA), equipped with a turbo ion spray interface. A Kinetex C18 column (150 × 4.6 mm; 2.6 µm particle size) protected by a Kinetex UHPLC Ultra C18 guard column (0.5 µm porosity × 4.6 mm inner diameter; Kinetex, Phenomenex, Torrance, CA, USA) was used. The gradient elution and optimized parameters were adapted from [55]. The optimized parameters for IAA and its precursors were obtained from the Analyst software (v 1.6.3) and aligned with the proposed pathways for IAA synthesis in plant growth-promoting bacteria [56].

#### 2.2.2. Quantitative Testing of Phosphate Solubilization

Flasks containing Pikovskaya medium supplemented with tricalcium phosphate (Ca_3_P_4_O_8_) as a P source were inoculated and incubated at 30 °C and 130 rpm with continuous shaking (SEV, Model 6090, Washington, DC, USA) [57]. Then, 15 mL of the bacterial inoculants were centrifuged in 50 mL conical tubes at 10,000 rpm for eight minutes (Eppendorf, Model 5804 R, Hamburg, Germany) [58]. Next, 2 mL of the supernatant were mixed with 2 mL of reagent (1:1 *v*/*v*) and left to react for one hour. Absorbance was measured at 882 nm using a spectrophotometer (Jenway, Model 6305, London, UK). The intensity of the blue color was correlated with the amount of P solubilized by the bacterial strains [59]. The kinetics of phosphate solubilization by the strains were evaluated on days 0, 5, 10, and 15 after incubation using the molybdenum blue method to analyze their performance [57,60].

#### 2.2.3. Acetylene Reduction Assay (ARA)

First, 50 µL of pure culture were inoculated into nitrogen-free mannitol semisolid agar in 100 mL vials, which were then sealed with rubber stoppers and incubated for 48 h at 28 °C. Vials showing bacterial growth were analyzed for acetylene reduction. Up to 10% of the vial’s atmosphere was replaced with acetylene (C_2_H_2_) [61]. The flame ionization detector and injector were set to 230 °C, and an HP-INNOWax Columns (Agilent, Santa Clara, CA, USA) was used at 100 °C with a 20 min cycle. The nitrogen gas flow was adjusted to 80 psi [39,61].

#### 2.2.4. Siderophore Production

The ability of strains to produce siderophores was qualitatively evaluated using chrome azurol agar (CAS) [53,62,63]. Plates containing CAS agar were inoculated and incubated at 28 ± 2 °C for 48–72 h. The presence of yellow-orange halos around the colonies on blue agar was indicative of siderophore excretion [53].

#### 2.2.5. Amplification of ACC Deaminase and Some Antimicrobial Compounds’ Genes

Genomic DNA was extracted using the ZR Soil Microbe DNA Kit Miniprep™ (Zymo Research, Orange, CA, USA), following the manufacturer’s instructions. Polymerase chain reaction (PCR) amplifications were performed in 50 µL reaction volumes containing 1× PCR buffer (5 µL per tube), MgCl_2_, (1.5 µL), 5 µL of each primer, dNTP (1 µL), Taq DNA polymerase (0.25 µL; Invitrogen, São Paulo, Brazil), and template DNA from each strain (1 µL). The *acdS* gene amplification and fungal antibiotic-producing genes oligonucleotides were selected based on protocols from [64,65,66,67,68] (Table 1). Oligonucleotides were supplied by T4 Oligo, with the manufacturer’s analysis certificate. The cycles were run on a thermal cycler (iCycler Thermal Cycler Firmware v 4.006, Bio-Rad Laboratories, Inc., Hercules, CA, USA) under the following conditions: (i) Initial denaturation at 95 °C for 3 min; (ii) Denaturation at 94 °C for 45 s; primer annealing temperatures were set according to [68], with 30-s annealing for all primers; (iii) Extension at 72 °C for 3 min (30 cycles) and final extension at 72 °C for 10 min. Agarose gel electrophoresis (1%) was performed in a horizontal gel box (ENDURO™ 7.10, Labnet International, Inc., Edison, NJ, USA), using a power supply (PowerPac HC High-Current Power Supply, Bio-Rad Laboratories, Inc.) at 120 volts for 40 min in 1× TBE buffer (Thermo Fisher Scientific Inc., Waltham, MA, USA), diluted in deionized water filtered twice with a 20 µm membrane. Bands were visualized under UV transillumination using the Gel Doc 2000 system (Bio-Rad Laboratories, Inc.).

**Table 1:** Targeted genes and their corresponding primers and sequences used in this research.

Gene	Primer	Primer Sequence	Melting Temp (°C)	Putative Gene Function	Amplicon Size (bp)	Reference
*prn*D	PRND1	GGGCGGGCCGTGGTGAT	65	Pyrrolnitrin biosynthesis enzyme	786	[65]
	PRND2	GGACGCSGCCTGYCTGGTCTG				
*phl*D	B2BF	ACCCACCGCGCATCGTTTATGAGC	66.5	Polyketide synthase III immediate precursor to 2,4-diacetylphloroglucinol	629	[64]
	BPR4	CCGCCGGTATGGAAGATGAAAAAGTC				
*phz*F	Ps_up 1	ATCTTCACCCCGGTCAACG	57	Phenazine biosynthesis enzyme	427	[67]
	Ps_low 1	CCRTAGGCCGGTGAGAAC				
*plt*C	PLTC1	AACGATCGCCCCGGTACAGAACG	58	Polyketide synthase I (Pyoluteorines)	438	[65]
	PLTC2	AGGCCCGGACACTCAAGAAACTCG				
*acd*S	F1936f	GCTCCTACTCTGTCACCTATCGHGAMGACTGCAAYWSYGGC	50	Gene encoding ACC deaminase	792	[68]
	F1938r	CTGTCGCTCTGGCTGTCACATVCCVTGCATBGAYTT				

### 2.3. Phylogenetic Analysis

DNA extraction was carried out using the Quick-DNA Miniprep Plus kit (Zymo Research, Irvine, CA, USA). The amplification of the 16S rDNA gene was performed using the universal primers, 27F (forward) 5′-AGA GTT TGA TCM TGG CTC AG-3′ and 1492R (reverse) 5′-CGG TTA CCT TGT TAC GAC TT-3′, and following the methodology described in previous studies [69]. Sequence editing, assembly, and comparison using the BLAST (Basic Local Alignment Search Tool, v 2.9.0) program against the National Center for Biotechnology Information (NCBI) database (www.ncbi.nlm.nih.gov) were performed. The sequences were compared with those reported in the database, and the sequences with the highest similarity were selected for phylogenetic analysis [17]. A multiple alignment of all selected sequences was performed on the phylogeny.fr platform with the following steps: Sequences were aligned using multiple sequence comparison by log-expectation computer program (MUSCLE; v3.8.31; multiple sequence alignment with high accuracy and high throughput) configured for maximum accuracy (default MUSCLE settings). After alignment, ambiguous regions were removed using Gblocks (v0.91b). A phylogenetic tree was reconstructed using the phylogenetic estimation maximum likelihood method in the PhyML software package (v3.1/3.0 aLRT). The HKY85 substitution model was selected, assuming an estimated proportion of invariable sites (0.836) and 4 gamma-distributed rate categories to account for rate heterogeneity among sites. The gamma shape parameter was directly estimated from the data (gamma = 0.481). The reliability of the internal branches was assessed using the aLRT (approximate likelihood ratio test, SH-like) method. The graphical representation and editing of the phylogenetic tree were done with TreeDyn (v198.3) [70,71,72,73,74].

### 2.4. Selection of Bacterial Strains for Bioassay and Preliminary Screening

Strains that exhibited at least one mechanism of action and had a good yield in the mechanism of action tests were selected for the bioassay. Additionally, a previous test with *Pinus radiata* seedlings was performed, where germinated seeds, germination speed, and root elongation were evaluated. Therefore, sixteen strains that were isolated from the rhizosphere and endophyte regions were selected for evaluation in *P. montezumae* and *P. patula* seeds. This was a screening to identify strains with better yields.

### 2.5. Adherence Assay

The seeds were placed in a container with cold water for 48 h, and empty seeds were discarded. The seeds were stratified at temperatures between 2 and 5 °C for 4 to 5 weeks as a pre-germination treatment to break physiological dormancy [75]. The seeds were then surface sterilized with 5% NaClO for 5 min, followed by three consecutive washes with sterile distilled water [76,77]. The sterility of the surface-sterilized seeds was confirmed by placing a few seeds in contact with a gelified medium. A microbial suspension was prepared in water following the protocol by [51]. For the adhesion assay, the protocol described by [69] was followed. The adhesion of 16 strains was tested, with four replicates for each one. The inoculated seeds were placed in a nursery under a 12/12 h light/dark photoperiod at 25 °C. The control seeds were immersed in sterile distilled water following the same protocol as the inoculated seeds.

### 2.6. Speed Germination Assay

Germination rate was examined based on the time elapsed after the seeds were inoculated and sown in sterile vermiculite, with a seed considered germinated once the radicle had emerged. Counts of the number of germinated seeds were conducted every six days, including only seeds with emerged radicles. The Germination Speed Index (GSI) was calculated according to [78].

### 2.7. Seedlings Inoculation

Twelve treatments with four replicates for *P. montezumae* seedlings and fifteen treatments with four replicates for *P. patula* seedlings were performed. The strains were selected for their highest yields in the germination speed and seed adhesion tests. The seedlings were transplanted into forest soil (vitric Andosol T-4), mixed with vermiculite in a 60/40 ratio. The soil was sterilized in two cycles of four hours, each at 120 °C, using a semi-industrial autoclave (Prendo Mod. AH80170, SEV-Prendo, Puebla, México). The mixture was placed in plastic containers of 1 L capacity to transplant the seedlings of both species into each container. The seedlings were inoculated weekly for one month with 2 mL of microorganism suspension in water [17]. Following this period, inoculations were carried out every two weeks for two months. The seedlings were subjected to water stress to evaluate their response to drought conditions. This qualitative method involved withholding irrigation for two weeks. Observations focused on how the seedlings responded and adapted to these adverse conditions, providing valuable insights into their drought resistance and survival mechanisms.

The plants were harvested 100 days after the experiment began. The tested variables included height, diameter, root length, and number of roots. Data were analyzed using InfoStat 2020 version [79] to perform analysis of variance (ANOVA) and Duncan’s multiple range test, with a significance level of α = 0.05.

## 3. Results

### 3.1. Isolation of Bacterial Strains and Assessment of Seedling and Soil Conditions

A total of 102 bacterial strains were isolated from *P. montezumae* and *P. patula* seedlings. Notably, 30 strains were recovered from *P. montezumae*, with 17 classified as rhizospheric and 13 as endophytes. In contrast, 57 strains were isolated from *P. patula*, 41 from the rhizosphere and 16 as endophytes. This demonstrates a diverse bacterial community associated with both the roots and internal tissues of these species. The differences in the number of strains between the two pines may be linked to the specific soil characteristics and environmental conditions at each collection site (Table 2).

### 3.2. Detected Growth-Promoting Mechanisms in Bacterial Isolates

The intracellular production of IAA via the indolepyruvic acid (IPyA) pathway, which is a tryptophan-dependent pathway, was identified. This was observed in 16 of the evaluated strains, with IAA production ranging from 1 to 305 μg/mL. Results indicated that most strains predominantly synthesize IAA through the IPyA pathway. However, the strain C63STPp was noted to preferentially utilize the tryptamine (TAM) and indoleacetamide (IAM) pathways (Table 3), suggesting variability in the metabolic routes employed by the strains for IAA production.

A diazotrophic bacterial biofilm was observed to be forming in the medium’s subsurface after 7 days of incubation at 30 °C. The biofilm was visible within the first 1–3 days post-inoculation, and daily growth was monitored since some bacteria exhibit accelerated growth rates. Among the evaluated strains, eleven demonstrated acetylene-reducing activity. Six were isolated from the rhizosphere and five were endophytes, with the reduction ranges between 8% and 29% (Table 3). Samples from positive strains were collected 96 h after injecting 10% acetylene into the vial atmosphere to assess their performance over time. However, an 80% decrease in performance was noted during this period, indicating that strains exhibit greater efficiency 48 h after injection. This suggests an optimal window for peak acetylene-reducing activity in diazotrophic strains.

Twenty-six strains tested were positive for phosphorus solubilization using the ammonium molybdate and ascorbic acid method, with phosphorus solubilization concentrations ranging from 0.1 to 2.4 mg/L. Under these incubation conditions, strain C12M*Pm* C12M*Pm* was the most efficient in phosphate solubilization, followed by strains C42ST*Pp*, C44ST*Pp*, C21M*Pm*, and C40ST*Pp*, in order of efficiency (Table 3). The maximum strain yield was observed between days 5 and 10 of incubation, where exponential phosphorus solubilization occurred. After day 10, performance declined. However, three strains initially showed a modest performance during the first five days, with a significant increase from the sixth day, peaking on day 10.

Twelve strains were identified as siderophore producers based on the formation of orange halos around their colonies. Eleven were isolated from the rhizosphere and one was classified as endophytic. Specifically, five strains were associated with *Pinus montezumae* and seven with *Pinus patula* (Table 3).

### Amplification of ACC Deaminase and Antimicrobial Compounds Genes

The strains C13M*Pm*, C28M*Pm*, and C38ST*Pp* showed amplification of the *prnD* gene, which is responsible for the synthesis of pyrrolnitrin (Table 4). This suggests their potential in producing this antimicrobial metabolite. Additionally, molecular analysis revealed that the strain C25M*Pm*, isolated from the rhizosphere of *Pinus patula*, had a positive amplification of the *acdS* gene, which encodes the ACC deaminase enzyme (Table 4). The presence of this gene indicates the strain may play a crucial role in reducing ethylene stress in plants, enhancing their growth and resilience under adverse conditions.

### 3.3. Molecular Identification of Strains and Their Phylogenetic Comparison

**Figure 1:**
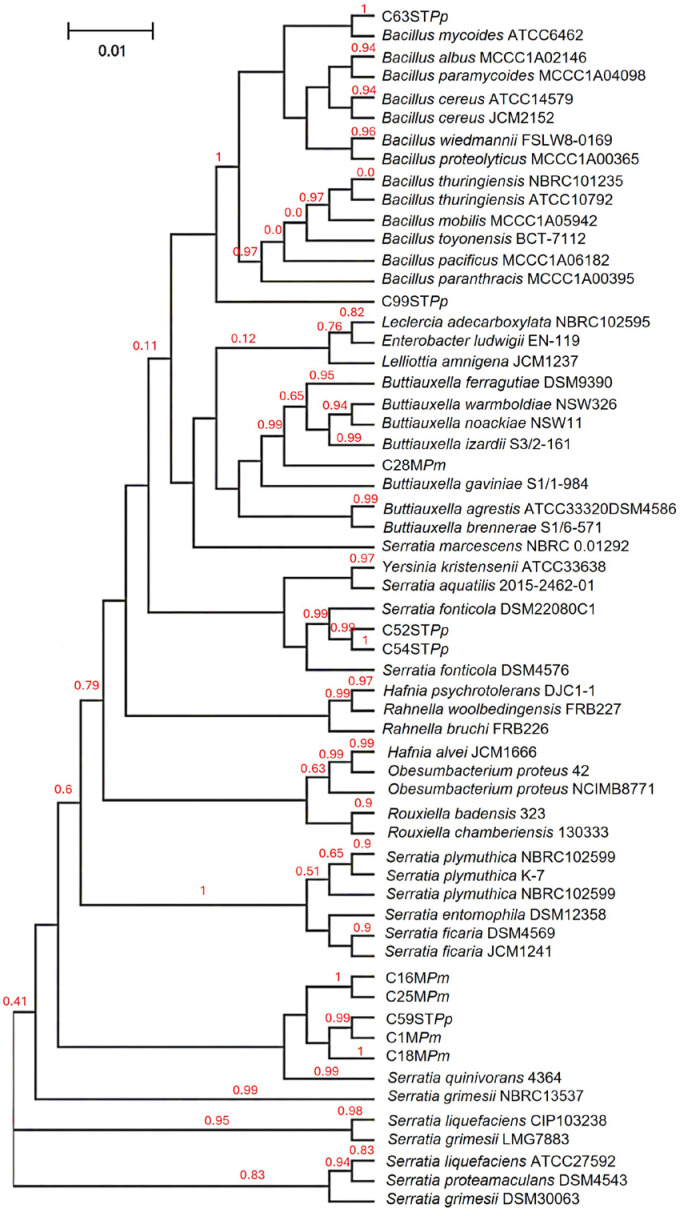
Phylogenetic tree based on 16S rDNA gene sequencing, the evolutionary relationship between the 10 growth-promoting isolates inferred using the Phylogeny platform is observed. Evolutionary distances were computed using the maximum likelihood method.

Ten strains were identified as part of the group with potential plant growth-promoting abilities. These strains are closely related to the genera *Serratia* (C1M*Pm*, C13M*Pm*, C16M*Pm*, C18M*Pm*, C25M*Pm*, C52ST*Pp*, C54ST*Pp*, C59ST*Pp*), *Buttiauxella* (C28M*Pm*), and *Bacillus* (C63ST*Pp*, C99ST*Pp*). A phylogenetic analysis of the identified strains, using sequences from bacteria related to these genera and considering the habitat from they were isolated, revealed clustering into two distinct taxonomic groups—γ-proteobacteria and Bacilli (Figure 1). The nucleotide sequences for the strains were submitted to the GenBank database and assigned the following accession numbers: PQ435155 (C1M*Pm*), PQ435156 (C16M*Pm*), PQ435157 (C18M*Pm*), PQ435158 (C25M*Pm*), PQ435159 (C28M*Pm*), PQ435160 (C52ST*Pp*), PQ435161 (C54ST*Pp*), PQ435162 (C59ST*Pp*), PQ435163 (C63ST*Pp*), and PQ435164 (C99ST*Pp*).

### 3.4. Effect of Plant Growth-Promoting Bacteria on Biomass and Root Structure of Pine Seedlings

#### 3.4.1. Adherence and Colonization Assays

*Pinus montezumae* seeds germinated after 10 days, while *P. patula* seeds germinated after twelve days following inoculation and planting in sterile vermiculite. In contrast, the control group seeds germinated on day 20. The variability in germination times between the two pine species was notable. *P. montezumae* exhibited a germination range of two to four seeds per treatment, achieving an 89% success rate. In contrast, *P. patula* seeds reached a 98% germination rate across all treatments.

The seeds were inoculated with bacterial suspensions of 1 × 10⁹ and 6 × 10⁹ CFU/mL to seeds of *P. patula* and *P. montezumae*, respectively. The seedlings of both forest species, planted in sterile vermiculite, exhibited healthy growth, showing notable vigor. The surface sterility test confirmed that the seeds were free of microorganisms, as no growth was observed on the gelified medium. Additionally, bacterial adhesion was detected on the seeds of both species in each treatment, with CFU per seed values ranging from 2 × 10⁶ for strain C63ST*Pp* to 4 × 10⁶ for strain C13M*Pm* (Table 5).

#### 3.4.2. PGPB Effect on Pines Seedlings

**Figure 2:**
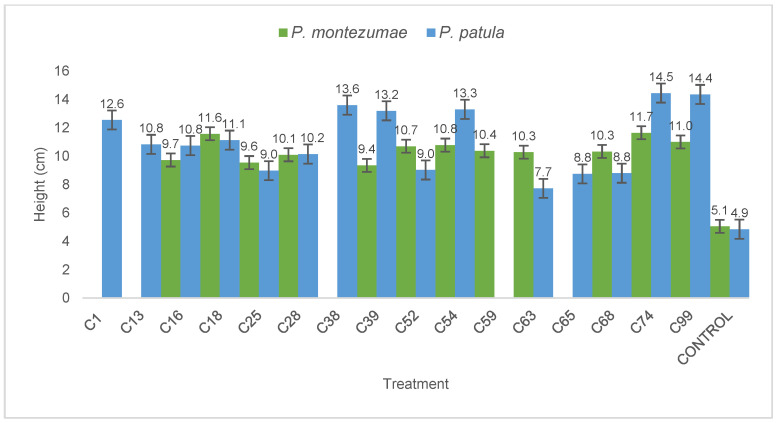
Evaluation of stem elongation in *P. montezumae* and *P. patula* seedlings after treatment with growth-promoting strains.

*Pinus montezumae* seedlings showed increased growth when inoculated with the rhizospheric strains C18M*Pm*, C52ST*Pp*, C54ST*Pp*, C74ST*Pp*, and C99ST*Pp*, (Figure 2). Strain C74ST*Pp*, isolated from *P. patula*, led to a seedling height increase of 11.7 cm. Regarding root length, five treatments resulted in elongation ranging from 26.1 to 32.1 cm, specifically with strains, C18M*Pm*, C28M*Pm*, C54ST*Pp*, C63ST*Pp*, and C74ST*Pp*. Additionally, treatments with the strains C52ST*Pp*, C54ST*Pp*, C59T*Pp*, C63ST*Pp*, C74ST*Pp*, and C99ST*Pp* showed increased root diameter (Table 6).

In terms of root number in seedlings, treatments with strains C16M*Pm*, C18M*Pm*, C28M*Pm*, C39ST*Pp*, C52ST*Pp*, C74ST*Pp*, and C99ST*Pp* demonstrated enhanced development, with a range of 9 to 10 roots per seedling. For *P. patula* seedlings, height growth ranged from 8.7 to 13.3 cm in the aerial parts. Treatments with strains C1M*Pm*, C38M*Pm*, C39ST*Pp*, C54ST*Pp*, C74ST*Pp*, and C99ST*Pp* showed significant post-inoculation growth, with the C74ST*Pp* strain being the most effective, achieving a height of 13.3 cm. The root length evaluation, seedlings from inoculated treatments showed the highest yield compared to control plants, with lengths ranging from 21.8 to 30.6 cm. The bacterial strains contributing to improved performance were C1M*Pm*, C18M*Pm*, C28M*Pm*, C52ST*Pp*, C65ST*Pp*, and C74ST*Pp*, significantly enhancing root development. For root diameter, treatments inoculated with the strains C16M*Pm*, C18M*Pm*, C38ST*Pp*, C39ST*Pp*, C74ST*Pp*, and C99ST*Pp* showed notable increases. Regarding root number, treatments with the strains C38ST*Pp*, C39ST*Pp*, C54ST*Pp*, C65ST*Pp*, C74ST*Pp*, and C99ST*Pp* resulted in a higher count, ranging from 10 to 11 roots per seedling (Table 6).

The treatments applied to *P. patula* seedlings showed a significant increase in height, with statistical differences supporting their enhanced yield in this aspect. On the other hand, in terms of root length and number, the treatments applied to *P. montezumae* seedlings were the most effective, showing significant differences compared to other treatments.

## 4. Discussion

In microorganisms, at least three Trp-dependent metabolic pathways for IAA biosynthesis have been identified, namely the IPyA pathway, the AIM pathway, and the TAM pathway [80,81]. Despite the diversity in prokaryotic metabolism, IAA biosynthesis predominantly follows two pathways, the IAM route and the IPyA route, excluding the TAM pathway and the Trp-independent pathway [82]. A characteristic pattern of IAA biosynthesis has been observed in plant–microbe interactions, specifically related to the ecophysiological role of the bacterial species involved, whether pathogenic or growth-promoting bacteria [83]. Numerous bacteria from the taxonomic classes α-Proteobacteria, β-Proteobacteria, δ-Proteobacteria, and Bacilli are known to produce this compound [84]. Previously, Trp-dependent IAA production was reported in the genus *Bacillus* in pine species [85,86]. The results of the IAA production test in the present work highlight the significant role of auxin-producing bacteria in promoting plant growth. The strains analyzed demonstrated the ability to produce indole-3-acetic acid (IAA), a crucial phytohormone involved in various plant developmental processes. This metabolic capability was detected across several bacterial strains, indicating their potential as PGPB. Furthermore, the metabolic pathway used by these strains aligns with previous studies [87,88,89,90], showing that both rhizospheric and endophytic bacteria can synthesize IAA, thereby enhancing seed germination and early seedling growth, which is particularly relevant for forest species like *Pinus* spp. These findings reinforce the importance of microbial auxin production in fostering mutualistic plant–microbe interactions and suggest a broader ecological role for these bacterial isolates in forest restoration and sustainable agriculture [91].

The ARA assay has been used to identify diazotrophic bacteria isolated from *P. patula*, including species such as *Bacillus macerans* and γ-proteobacteria (*Pseudomonas* sp.). A reduction in activity peak was observed after 3 h of evaluation, with reduction ranges varying between 110 and 120 nmol of acetylene [39]. The ARA results of our work revealed the presence of nitrogen-fixing capabilities in the bacterial strains associated with *Pinus patula*. These strains, including *Bacillus macerans* and *Pseudomonas* sp. [92,93,94,95], demonstrated significant asymbiotic nitrogen fixation activity, a crucial mechanism for enhancing plant nitrogen uptake. These findings emphasize the potential of these diazotrophic bacteria to contribute to nitrogen input in forest ecosystems, which can support the growth of *Pinus* species by supplementing essential nutrients, especially in nutrient-poor soils targeted for reforestation.

The evaluation of phosphorus-solubilizing bacteria (PSB) varies over time; for instance, maximum phosphate solubilization is typically achieved after 3 days of incubation. However, extending incubation to 5 days does not further improve the solubilization extent [57]. In our study, some strains demonstrated notable efficiency in phosphate solubilization, with a decline in activity observed after 10 days of incubation, while others reached the peak activity on day 10. This variation may be related to their growth capacity or gene expression in the culture medium [38]. The primary mechanism of mineral phosphate solubilization involves the action of organic acids synthesized by PSB, along with the production of phosphatase enzymes [41,95]. Notable phosphate-solubilizing acids reported include gluconic and 2-ketogluconic acids, which are consistently identified in these bacteria [96]. Additionally, other organic acids with phosphate-solubilizing capacity are oxalic, citric, butyric, malonic, lactic, succinic, malic, acetic, fumaric, adipic, and indoleacetic acids [97,98]. There is a close link between acidic pH and effective phosphate solubilization; a decrease in pH clearly indicates acid production, which is considered responsible for phosphate solubilization. It is suggested that the microorganisms reducing the medium pH during growth are effective PSB [21]. The phosphate solubilization assays of this work revealed that several bacterial strains demonstrated significant potential as PSB, capable of performing such a critical function for plant growth promotion. These findings highlight the important role of PSB in enhancing nutrient availability for plants and contributing to improved growth, particularly in phosphorus-deficient soils.

The siderophore production assays performed in the present work demonstrated that 12 bacterial strains effectively produced these iron-chelating compounds, playing a crucial role in promoting plant growth and inhibiting pathogens [41,99]. The CAS assay revealed strong siderophore activity, indicated by the color change from blue to orange, confirming the ability of the strains to sequester ferric iron [100]. This process enhances iron availability for plants, particularly in iron-deficient environments, while simultaneously limiting access to iron for harmful microorganisms [101]. The results underscore the potential of these strains to improve plant resilience by enhancing nutrient uptake and protecting against pathogens, particularly in forest ecosystems.

PGPB exhibit beneficial properties for plants, linked to the expression of specific genes that enhance nutrient uptake and mitigate the negative effects of phytopathogens [102]. ACC deaminase and IAA production are crucial in plant–bacteria interactions due to their role in promoting root elongation [95]. The unexpected results of *acdS* gene amplification in our tests were surprising, as it was expected that IAA-producing strains would express this gene. The close relationship between enzyme production and plant growth regulators arises because ACC synthase, which converts S-adenosylmethionine (SAM) to ACC, is stimulated by these regulators [103]. The *acdS* gene, frequently identified in rhizobacterial genera from various soil types across different geographical areas, is co-regulated by the *acdR* and *acdB* proteins [104,105,106]. Microbial deamination of ACC reduces ethylene concentration, providing a beneficial mode of action for plants [107]. Additionally, strains C13M*Pm*, C28M*Pm*, and C38ST*Pp* amplified the *prnD* gene, associated with the synthesis of pyoluteorin, an antimicrobial involved in the biological control of soil pathogens [99]. There is a possibility that strains isolated from *P. montezumae* and *P. patula* may produce other antimicrobials for the biological control of phytopathogens. γ-proteobacteria are known for producing various antimicrobial compounds, such as 2,4-diacetylphloroglucinol (DAPG), pyoluteorin (PLT), pyrolnitrin (PRN), hydrogen cyanide (HCN), and gluconic and 2-ketogluconic acids, produced via the direct oxidation of glucose pathway [108].

Studies have demonstrated the potential of *Serratia* strains to promote plant growth through various mechanisms, including the following: phosphate solubilization; the production of indole-3-acetic acid (IAA) and 1-aminocyclopropane-1-carboxylate (ACC) deaminase; the synthesis of antimicrobial compounds, siderophores, and quorum-sensing molecules such as N-acyl homoserine lactones (AHLs); systemic resistance (ISR) against pathogens [109,110,111,112,113]; and increased drought stress tolerance [114,115]. Additionally, one study identified a strain of *Bacillus*, known for its genetic diversity and spore-forming ability, which is valuable in the production of stable bioinoculants [116,117]. Both *Serratia* and *Bacillus* strains have been documented in the literature for their roles in the isolation and growth promotion of pine seedlings. In this study, we present a table that contrasts our findings with those reported in the previous research, providing a comprehensive comparison of the effects of these strains on pine seedling growth (Table 7). This genus, widely distributed in many ecological niches, is frequently isolated and is remarkable for wide applications in ecology, biotechnology, industry, and clinical microbiology, with important research conducted regarding its genetic diversity [118]. *Buttiauxella* is a genus with limited reports on its role as a plant growth promoter. One of the investigation reports [119] described an endophytic strain identified as *Buttiauxella* sp. SaSR13, which demonstrated successful colonization in the root elongation zone that was attributed to increased IAA concentrations and reduced superoxide anion levels, along with improvements in the root exudates, particularly malic and oxalic acids in *Sedum alfredii*. This resulted in significant growth enhancement and cadmium accumulation. Conversely, other studies [120,121] have shown that the *Buttiauxella* strains isolated from the rhizospheres of *Festuca arundinacea* and *Vaccinium* spp. are highly effective in solubilizing inorganic phosphorus, exhibiting catalase activity, and producing organic acids and siderophores. In our research, a *Buttiauxella* strain associated with *P. montezumae* was characterized, demonstrating its capability to promote the growth of both *P. patula* and *P. montezumae*, suggesting a similar beneficial potential as the other beneficial bacteria.

The speed of seed germination is critical for seedling growth under adverse conditions, reducing the risk of phytopathogenic infections or latent infections following transplantation [122,123]. Evidence supports that faster and more uniform germination leads to a significant reduction in seedling mortality and an increase in the number of viable seedlings [124,125,126]. While increased shoot height due to bacterial inoculation may not be essential, the health and architecture of the root system are crucial to successful development and transplant survival. These factors greatly influence the survival post-transplantation [47,75]. However, the effect of bacteria on shoot and seedling growth appears to be species-specific and independent. PGPB play a vital role in enhancing seedling growth in nurseries, as assessed through established biometric parameters such as stem length, collar diameter, and dry weight [106]. Our results for shoot height and root length are consistent with previous research in forest species inoculated with bacterial strains [17,39,47,127,128]. Although the duration of trials may vary, the evaluated strains show plant growth improvements, even in short-term trials. Strains isolated from the rhizosphere of *Pinus patula* in Colombia have also shown enhanced plant performance following inoculation [39]. The results of this study highlight the importance of seed germination speed in *Pinus* species to improve seedling growth under adverse conditions. The bacterial strains evaluated demonstrated improvements in seedling growth, confirming their potential as plant growth-promoting agents in forest species.

**Table 7:** Comparison of treatment yields with similar works.

Reference	Strains	Total Treatments	Forestry Species	Height Range (cm)	Root Length Range (cm)	Root Diameter Range (mm)	Duration of Trials (Months)
[47]	*Serratia marcescens*, *Bacillus subtilis*, *Paenibacillus macerans*, *Bacillus pumilus*, *Bacillus sphaericus*	12	*Pinus taeda* L. and *Pinus elliottii* (Engelm.)	13–14.8	15.6–17.9		3
[128]	*Enterobacter intermedius*, *Pseudomonas fluorescens*, *Chryseobacterium balustinum*, *Phosphorobacillus latus*	4	*Quercus ilex* ssp. Ballota and *Pinus pinea*	17.52–19.72			4
[127]	*Bacillus* sp., *Curtobacterium* sp., *Arthrobacter* sp., *Staphylococcus* sp., *Burkholderia* sp.	10	*Pinus pinea*	20–26			5
[39]	*Pseudomonas* sp., *Bacillus macerans*., *Enterobacter agglomerans*., *Suillus luteus*., *A. chroococcum*.	11	*Pinus patula*	7.3–18.7			12
[17]	*Cupriavidus basilensis*., *Rhodococcus qingshengii*., *Pseudomonas* spp., *Pseudomonas gessardii*., *Stenotrophomonas rhizophila*., *Rhodococcus erythropolis*., *Cohnella* sp.	10	*Pinus pseudostrobus* (Lindl.).	4.6–6.6			5
This study	*Serratia* sp., *Buttiauxella* sp., *Bacillus* sp.	16	*Pinus montezumae* and *Pinus patula*	7.7–14.5	17.5–29.5	1–2	4

The ANOVA test revealed significant differences in the growth responses of the *Pinus patula* and *Pinus montezumae* seedlings to the treatments applied, highlighting specific patterns for each species. The most notable outcome for *P. patula* was the significant increase in seedling height, indicating that the applied treatments had a clear and statistically significant effect on this growth parameter. This suggests that the factor being tested, likely the inoculation conditions, played a crucial role in promoting height in *P. patula* seedlings. In contrast, *P. montezumae* seedlings showed better performance in terms of root length and the number of roots, although these differences were not statistically significant in the other growth parameters. This indicates that while the treatments had a positive effect on the root system of *P. montezumae*, this effect was not strong enough to reflect significant changes in additional traits, such as stem height or overall biomass. Thus, the treatments appear to be more effective in influencing root morphology in *P. montezumae*, but further refinement may be required to observe broader impacts. Interestingly, no significant differences were observed in root diameter for either species. This suggests that root diameter might be less sensitive to the treatments applied, or that other factors, such as genetic traits play a more dominant role in determining this particular parameter. The lack of statistical significance for root diameter may also imply that the methods used need further optimization to influence this trait. From these observations, several perspectives emerge. First, the treatments could be optimized to achieve more consistent results across all growth parameters. While height and root length responded positively in each species, focusing on improving other traits, like root diameter, could lead to more comprehensive growth enhancements. Additionally, further exploration of species-specific responses could provide insights into why *P. patula* responded better in terms of height, while *P. montezumae* showed more robust root growth. Moreover, incorporating additional variables beyond height and root traits, such as photosynthetic rate, nutrient uptake, or stress tolerance, could offer a more complete picture of how the treatments affect plant health and development. This would also provide valuable information for refining treatment protocols in both nursery and field conditions. These findings could have significant implications for reforestation and forest management programs. By tailoring treatments to enhance specific traits, such as height in *P. patula* for timber production or root growth in *P. montezumae* for soil stabilization, can improve the success rates of these programs.

This study highlights the significant role of PGPB in promoting growth and improving resilience in forest species such as *P. montezumae* and *P. patula*. Key mechanisms include phosphate solubilization, siderophore production, nitrogen fixation, and the synthesis of indole-3-acetic acid (IAA). These bacterial activities enhance nutrient availability, stimulate root development, and improve seed germination, which are crucial for successful reforestation, especially in nutrient-deficient soils. The potential application of these bacteria in forest restoration demonstrates their ecological importance and offers a promising approach to sustainable forest management. Further work will be needed to demonstrate that these mechanisms are indeed associated with the growth promotion we have observed in situ. However, it is likely that several of the growth-promoting mechanisms observed in vitro for the bacteria explored in this study are also occurring in association with pine plants, as reported in other works [129,130].

## 5. Conclusions

This study identified and characterized the key mechanisms by which isolated strains from *Pinus patula* and *Pinus montezumae* promote plant growth. The evaluated strains exhibited plant growth-promoting mechanisms, including auxin production, phosphate solubilization, and siderophore production. Ten strains with the potential to enhance pine growth were selected for further molecular characterization, with seven belonging to the genus *Serratia*, one to *Bacillus*, and notably, one to the less commonly associated genus *Buttiauxella*. These findings suggest a promising approach to enriching soil microbial populations in reforestation efforts by utilizing nursery-grown plants inoculated with beneficial microorganisms. By introducing plants with a robust rhizospheric microbiota, not only is the reintroduction of beneficial microbes into the soil facilitated, but early plant growth is also enhanced, mitigating stress and leading to better soil adaptation compared to the non-inoculated plants.

## Figures and Tables

**Table 2:** Characteristics of sampled trees and bacterial strain isolation in different forest regions.

Tree Sample	Tree Species	Height (cm)	Soil Moisture %	Altitudinal Profile (masl)	Sampling Coordinates	Forested Region	Number of Isolated Strains
1	*P. montezumae*	20	60	2885	19°14′49″ N; 98°05′44″ O	Malinche National Park	4
2	*P. montezumae*	25	63	2962	19°15′02″ N; 98°05′22″ O		5
3	*P. montezumae*	26	68	3030	19°15′03″ N; 98°05′2″ O		6
4	*P. montezumae*	30	56	3077	19°15′04″ N; 98°05′21″ O		5
5	*P. montezumae*	46	61	2896	19°15′01″ N; 98°05′20″ O		3
6	*P. montezumae*	32	54	3061	19°15′17″ N; 98°04′55″ O		4
7	*P. montezumae*	56	53	2931	19°15′51″ N; 98°05′23″ O		3
8	*P. patula*	35	53	2923	19°41′31″ N; 98°04′43″ O	Sierra de Tlaxco-Caldera-Huamantla	22
9	*P. patula*	58	51	2853	19°41′35″ N; 98°04′44″ O		16
10	*P. patula*	26	54	2903	19°41′34″ N; 98°04′43″ O		19

**Table 3:** Growth-promoting activity and origin and identification of bacterial strains isolated from *P. patula* and *P. montezumae*. (R = Rhizospheric; E = Endophytic; missing data indicate that no detectable growth-promoting activity was observed for the respective strain in the tests performed).

	Indole Test					
Strain	µg/mL Intracelular	Metabolic Pathway	Siderophores	P Solubilizing	ARA %	Substrate
C1M*Pm*	137	IPyA	+	-	-	R
C13M*Pm*	1	IPyA	-	1.5	13	R
C16M*Pm*	186	IPyA	+	1.5	-	R
C18M*Pm*	285	IPyA	+	1.7	-	R
C25M*Pm*	4	IPyA	+	0.4	-	R
C28M*Pm*	189	IPyA	-	-	-	E
C38ST*Pp*	4	IPyA	+	0.9	-	E
C39ST*Pp*	82	IPyA	+	2	-	R
C52ST*Pp*	78	IPyA	+	-	-	R
C54ST*Pp*	305	IPyA	+	2.6	-	R
C59ST*Pp*	88	IPyA	+	-	-	R
C63ST*Pp*	1	TAM IAM	-	0.7	-	R
C65ST*Pp*	2	IPyA	+	-	15	R
C68ST*Pp*	2	IPyA	+	-	-	R
C74ST*Pp*	110	IPyA	+	-	74	R
C99ST*Pp*	95	IPyA	-	-	-	E

**Table 4:** Amplification of genes with antagonistic effects, amplification of the ACC deaminase gene, and origin of bacterial strains isolated from *P. patula* and *P. montezumae* (R = Rhizospheric; E = Endophytic).

Strain	Genus	*prnD*	*phlD*	*phzF*	*pltC*	*acdS*	Substrate
C1M*Pm*	*Serratia* sp.	-	-	-	-	-	R
C13M*Pm*	N/D	+	-	-	-	-	R
C16M*Pm*	*Serratia* sp.	-	-	-	-	-	R
C18M*Pm*	*Serratia* sp.	-	-	-	-	-	R
C25M*Pm*	*Serratia* sp.	-	-	-	-	+	R
C28M*Pm*	*Buttiauxella* sp.	+	-	-	-	-	E
C38ST*Pp*	N/D	+	-	-	-	-	E
C39ST*Pp*	N/D	-	-	-	-	-	R
C52ST*Pp*	*Serratia* sp.	-	-	-	-	-	R
C54ST*Pp*	*Serratia* sp.	-	-	-	-	-	R
C59ST*Pp*	*Serratia* sp.	-	-	-	-	-	R
C63ST*Pp*	*Bacillus* sp.	-	-	-	-	-	R
C65ST*Pp*	N/D	-	-	-	-	-	R
C68ST*Pp*	N/D	-	-	-	-	-	R
C74ST*Pp*	N/D	-	-	-	-	-	R
C99ST*Pp*	*Bacillus cereus*	-	-	-	-	-	E

**Table 5:** Results of inoculation, seed adhesion, and germination rate assays for *P. montezumae* and *P. patula*. The treatment number corresponds to the identification number assigned to the isolated strains.

Treatment	UFC/mL Inoculate	UFC/Seed	Germination Speed Index (GSI)		Germinated Seeds	
			*P. montezumae*	*P. patula*	*P. montezumae*	*P. patula*
C1M*Pm*	6 × 10^9^	2 × 10^8^	0.306	0.264	3 ± 0.25	4 ± 0
C13M*Pm*	6 × 10^9^	2 × 10^7^	0.278	0.278	3 ± 0.25	4 ± 0
C16M*Pm*	6 × 10^9^	2 × 10^8^	0.472	0.278	4 ± 0	4 ± 0
C18M*Pm*	6 × 10^9^	2 × 10^8^	0.556	0.417	4 ± 0	4 ± 0
C25M*Pm*	6 × 10^9^	2 × 10^7^	0.194	0.389	3 ± 0.25	4 ± 0
C28M*Pm*	6 × 10^9^	2 × 10^9^	0.306	0.264	4 ± 0	4 ± 0
C38ST*Pp*	6 × 10^9^	2 × 10^9^	0.306	0.306	3 ± 0.25	4 ± 0
C39ST*Pp*	6 × 10^9^	2 × 10^8^	0.417	0.389	4 ± 0	4 ± 0
C52ST*Pp*	6 × 10^9^	2 × 10^7^	0.417	0.333	4 ± 0	4 ± 0
C54ST*Pp*	6 × 10^9^	2 × 10^7^	0.250	0.278	3 ± 0.25	4 ± 0
C59ST*Pp*	6 × 10^9^	2 × 10^9^	0.250	0.194	3 ± 0.25	3 ± 0.25
C63ST*Pp*	6 × 10^9^	2 × 10^8^	0.417	0.250	4 ± 0	4 ± 0
C65ST*Pp*	6 × 10^9^	2 × 10^6^	0.306	0.264	4 ± 0	4 ± 0
C68ST*Pp*	6 × 10^9^	2 × 10^7^	0.306	0.306	3 ± 0.25	4 ± 0
C74ST*Pp*	6 × 10^9^	2 × 10^9^	0.500	0.361	4 ± 0	4 ± 0
C99ST*Pp*	6 × 10^9^	2 × 10^7^	0.417	0.417	4 ± 0	4 ± 0
Control	-	-	0.097	0.097	2 ± 0.3	2 ± 0.3

**Table 6:** Results of the measured parameters of *P. montezumae* and *P. patula* seedlings after 100 days in nursery.

Strain	Height (cm)		Root Length (cm)		Root Diameter (mm)		Number of Roots	
	*P. montezumae*	*P. patula*	*P. montezumae*	*P. patula*	*P. montezumae*	*P. patula*	*P. montezumae*	*P. patula*
C1M*Pm*		12.6 ± 0.9 ^abc^		27.7 ± 0.9 ^a^		1 ± 0.02 ^bc^		8 ± 0.5 ^bcde^
C13M*Pm*		10.8 ± 0.9 ^bc^		23.5 ± 0.9 ^a^		1 ± 0.02 ^abc^		7 ± 0.6 ^cdef^
C16M*Pm*	9.7 ± 0.7 ^ab^	10.8 ± 0.6 ^bc^	23.7 ± 0.9 ^abc^	25.1 ± 0.9 ^a^	1 ± 0.03 ^ab^	2 ± 0.01 ^ab^	9 ± 0.6 ^abcd^	9 ± 0.5 ^abcde^
C18M*Pm*	11.6 ± 0.8 ^a^	11.1 ± 0.9 ^abc^	32.1 ± 0.9 ^bc^	26.2 ± 0.9 ^a^	1 ± 0.01 ^ab^	2 ± 0.02 ^ab^	10 ± 0.3 ^ab^	6 ± 0.9 ^def^
C25M*Pm*	9.6 ± 0.6 ^bcd^	9.0 ± 0.7 ^bcd^	17.5 ± 0.9 ^c^	22.9 ± 0.9 ^a^	1 ± 0.01 ^ab^	1 ± 0.02 ^bc^	7 ± 0.9 ^abcd^	7 ± 0.3 ^cdef^
C28M*Pm*	10.1 ± 0.4 ^ab^	10.2 ± 0.9 ^bc^	29.5 ± 0.9 ^ab^	27.0 ± 0.9 ^a^	1 ± 0 ^ab^	1 ± 0.02 ^abc^	9 ± 0.9 ^abcd^	9 ± 0.7 ^abcde^
C38ST*Pp*		13.6 ± 0.6 ^ab^		23.2 ± 0.9 ^a^		2 ± 0.01 ^ab^		11 ± 0.7 ^ab^
C39ST*Pp*	9.4 ± 0.4 ^b^	13.2 ± 0.6 ^abc^	24.8 ± 0.9 ^abc^	21.8 ± 0.9 ^a^	1 ± 0.01 ^ab^	2 ± 0.02 ^ab^	9 ± 0.5 ^abc^	11 ± 0.6 ^abc^
C52ST*Pp*	10.7 ± 0.9 ^ab^	9 ± 0.8 ^bcd^	19.9 ± 0.9 ^bc^	26.4 ± 0.9 ^a^	2 ± 0.03 ^a^	2 ± 0.01 ^ab^	9 ± 0.5 ^abcd^	6 ± 0.3 ^ef^
C54ST*Pp*	10.8 ± 0.5 ^ab^	13.3 ± 0.9 ^abc^	26.1 ± 0.9 ^abc^	22.1 ± 0.9 ^a^	2 ± 0.02 ^a^	2 ± 0.02 ^ab^	6 ± 0.5 ^abcd^	10 ± 0.5 ^abcd^
C59ST*Pp*	10.4 ± 0.4 ^ab^		19.7 ± 0.9 ^bc^		2 ± 0.03 ^a^		6 ± 0.8 ^bcd^	
C63ST*Pp*	10.3 ± 0.05 ^ab^	8.7 ± 0.4 ^cd^	26.3 ± 0.9 ^abc^	23.0 ± 0.9 ^ab^	2 ± 0.02 ^ab^	2 ± 0.01 ^ab^	6 ± 0.4 ^abcd^	9 ± 0.3 ^ef^
C65ST*Pp*		9 ± 0.9 ^bcd^		30.6 ± 0.9 ^a^		2 ± 0.01 ^ab^		11 ± 0.3 ^abc^
C68ST*Pp*	10.3 ± 0.8 ^ab^	9 ± 0.6 ^bcd^	25.2 ± 0.9 ^abc^	24.3 ± 0.9 ^a^	2 ± 0.02 ^a^	1 ± 0.01 ^abc^	8 ± 0.9 ^abcd^	6 ± 0.4 ^abcde^
C74ST*Pp*	11.7 ± 0.8 ^a^	16.7 ± 0.9 ^a^	28.7 ± 0.9 ^abc^	27.7 ± 0.9 ^a^	2 ± 0.04 ^a^	2 ± 0.01 ^a^	10 ± 0.9 ^a^	11 ± 0.6 ^a^
C99ST*Pp*	11.0 ± 0.4 ^ab^	13.3 ± 0.7 ^abc^	22.3 ± 0.9 ^abc^	23.5 ± 0.9 ^a^	2 ± 0.02 ^a^	2 ± 0.01 ^ab^	9 ± 0.8 ^abcd^	10 ± 0.6 ^abc^
Control	5.1 ± 0.6 ^c^	5 ± 0.8 ^d^	5.9 ± 0.9 ^d^	6.4 ± 0.8 ^b^	0.6 ± 0.01 ^b^	0.8 ± 0.03 ^c^	2 ± 0.8 ^e^	4 ± 0.9 ^fg^

Values with a common letter within each column are not significantly different according to Tukey's multiple comparison test at *p* ≤ 0.05.

## Data Availability

The raw data supporting the conclusions of this article will be made available by the authors on request.
